# Bibliometric and visualized analysis of peripartum respiratory complications from 2004 to 2023

**DOI:** 10.3389/fmed.2024.1395641

**Published:** 2024-10-29

**Authors:** Fan Ye, Teng Wang, Yijiao Chen, Fan Li, Xinyu Gu, Jie Xiao

**Affiliations:** ^1^Department of Anesthesiology, Renji Hospital, School of Medicine, Shanghai Jiaotong University, Shanghai, China; ^2^Key Laboratory of Anesthesiology (Shanghai Jiao Tong University), Ministry of Education, Shanghai, China

**Keywords:** peripartum respiratory complications, bibliometrics, visualization analysis, CiteSpace, risk factors

## Abstract

**Background:**

Peripartum respiratory complications are a major reason for pregnant women’s admission to the ICU, even endangers the maternal life. This bibliometric analysis was designed to elucidate the spectrum of diseases and risk factors for various respiratory complications during the peripartum period, as well as the future research directions in this field.

**Methods:**

Relevant publications were downloaded from the Web of Science Core Collection on November 1, 2023. CiteSpace was utilized for conducting the scientometric study.

**Results:**

A total of 2,331 articles and reviews on respiratory complications during pregnancy published between 2004 and 2023 were retrieved, comprising 87,244 co-cited references, originating from 568 institutions across 104 countries/regions. The United States emerged as the leading country in this domain, with Harvard University standing out as the most actively engaged institution. Bibliometric analysis reveals that the current research hotspots include “COVID-19 pandemic,” “venous thromboembolism,” “respiratory distress syndrome” and “cardiovascular diseases.” Meanwhile, “venous thromboembolism,” cytokine storm” and supportive management such as “extracorporeal membrane oxygenation” might represent potential future research directions.

**Conclusion:**

Over the past two decades, research on respiratory system complications in pregnancy has continually evolved. This study contributes to enabling researchers in the related field to understand future research hotspots and trends, providing information on potential collaborators, institutions, countries, and citation references.

## 1 Introduction

Peripartum respiratory complications constitute a complex set of multifactorial diseases, most commonly occurring during intrapartum and postpartum (1). They represent a significant factor for pregnant women requiring ICU admission and are a common cause of maternal mortality (2). Adaptive changes in the respiratory and circulatory systems during pregnancy render women susceptible to a range of respiratory complications, including asthma, pulmonary infections, pulmonary edema, pulmonary embolism, acute respiratory distress syndrome, with a more severe course compared to non-pregnant individuals (3). The hypercoagulable state in pregnancy increases the risk of venous thromboembolism fourfold, with subsequent pulmonary embolism remaining a major cause of morbidity and mortality in pregnant women in developed countries (4). Over the past two decades, there has been an increased incidence of severe perinatal pulmonary complications, notably represented by acute respiratory distress syndrome, with an overall incidence ranging from 15.9 to 130 cases per 100,000 deliveries (5). The viral pandemic of COVID-19 in 2019 has further exacerbated this trend. Scholars are increasingly focusing on the field of perinatal respiratory system complications, yet there remain numerous questions requiring further exploration in this area.

Bibliometrics employs quantitative and statistical methods to analyze existing scientific literature, facilitating comparisons of contributions from different countries, institutions, authors, and journals (6, 7). It is widely used to describe the current state and predict future trends in various medical fields. However, there is currently a lack of research in the field of respiratory system complications during pregnancy. Therefore, the aim of this study is to explore the research focus and frontiers in this area through bibliometric analysis, with the expectation of contributing to the formulation of future guidelines. Besides, we hope to investigate which underlying conditions may increase the risk of pregnant women developing respiratory complications, in order to enable early prevention and management in clinical practice. Furthermore, we hope to identify updated and safer effective treatment approaches for managing severe respiratory complications, thereby improving the overall safety of the pregnancy continuum from the antenatal to the postpartum period.

## 2 Materials and methods

### 2.1 Data acquisition and search strategy

**Figure 1:**
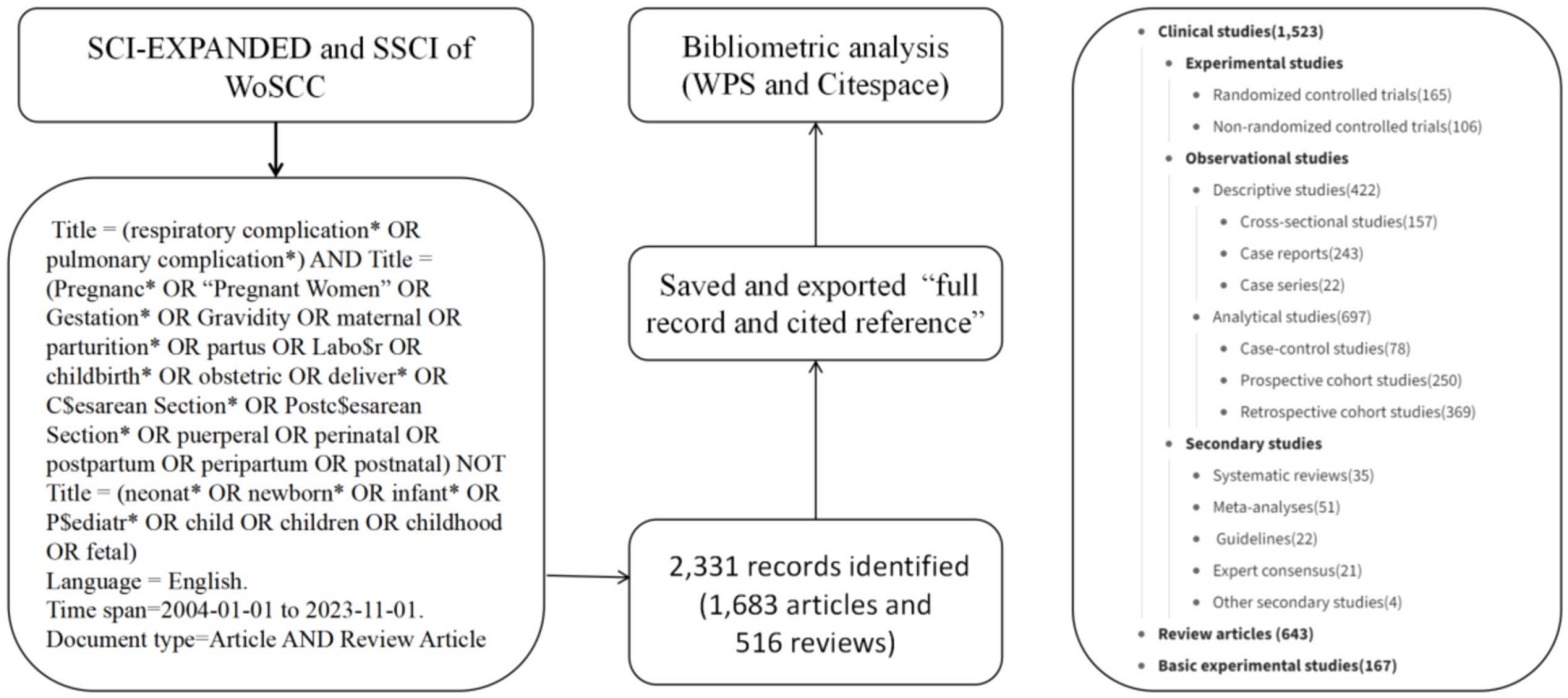
Data acquisition and search strategy.

The publications related to this research were downloaded from the Web of Science Core Collection (WoSCC) database, encompassing Social Science Citation (SCI)-EXPANDED and Social Sciences Citation Index (SSCI). The data was retrieved on November 1, 2023. Our search strategy is outlined as follows (Figure 1): Title = (respiratory complication* OR pulmonary complication*) AND Title = (Pregnanc* OR “Pregnant Women” OR Gestation* OR Gravidity OR maternal OR parturition* OR partus OR Labo$r OR childbirth* OR obstetric OR deliver* OR C$esarean Section* OR Postc$esarean Section* OR puerperal OR perinatal OR postpartum OR peripartum OR postnatal) NOT Title = (neonat* OR newborn* OR infant* OR P$ediatr* OR child OR children OR childhood OR fetal) AND Language = English.

The time span was set from January 1, 2004, to November 1, 2023. To enhance the precision of the database, less representative record types were filtered out, including proceedings papers (*n* = 98), early access (*n* = 30), editorial material (30), letter (*n* = 8), meeting abstract (*n* = 8), book chapters (*n* = 2), correction (*n* = 1), and retracted publication (*n* = 1). Consequently, the records were reduced to 2,331 original research articles (*n* = 1,683) and reviews (*n* = 516) (Figure 1). It is essential to note that search strategies of this nature inevitably include some irrelevant records. However, through analysis by CiteSpace, it may be possible to unveil areas relevant to the subject matter yet unrecognized by scholars, potentially leading to new discoveries (8).

### 2.2 Data collection

The WoSCC data were saved as a Plain Text File and exported in the format of “full record and cited reference,” including titles, authors, abstracts, journals, keywords, and references. Subsequently, the collected data were imported into CiteSpace V6.1.R6 Advanced (64-bit) (Drexel University, Philadelphia, PA, United States) for additional bibliometric analysis. Additionally, we calculated the impact factors (IF) of journals and literature, obtaining this information from the 2023 Journal Citation Report (Clarivate Analytics, Philadelphia, PA, United States).

### 2.3 Bibliometric analysis

CiteSpace is commonly employed for visualizing and analyzing literature, providing insights into the current state of research within a specific field and predicting potential future research areas. In our study, we utilized CiteSpace for literature visualization and analysis in five key areas: collaborative network analysis, keyword analysis, literature co-citation analysis, and journal co-citation analysis. And the annual publication trends and their respective categories were visualized using WPS.

Within the visualized network, each node represents a unique item, and its size is proportional to the quantity it represents (9). The color of the nodes corresponds to different time slice, providing a temporal dimension to the analysis (10). Nodes with outer purple circles indicate high betweenness centrality (more than 0.1), indicating their significant positions in the network. These nodes may denote pivotal moments in research or recent hotspots (10). The lines connecting nodes represent collaborative or co-citation relationships, revealing the interconnectedness within the research landscape.

The CiteSpace parameters were configured as follows: time slice (2004-01-01 to 2023-11-01), years per slice (1), term source (entire selection), g-index (*k* = 25), and selection criteria (top *N* = 50). Other parameters were maintained at their default settings.

## 3 Results

### 3.1 Annual growth trend of publications and their categories

**Figure 2:**
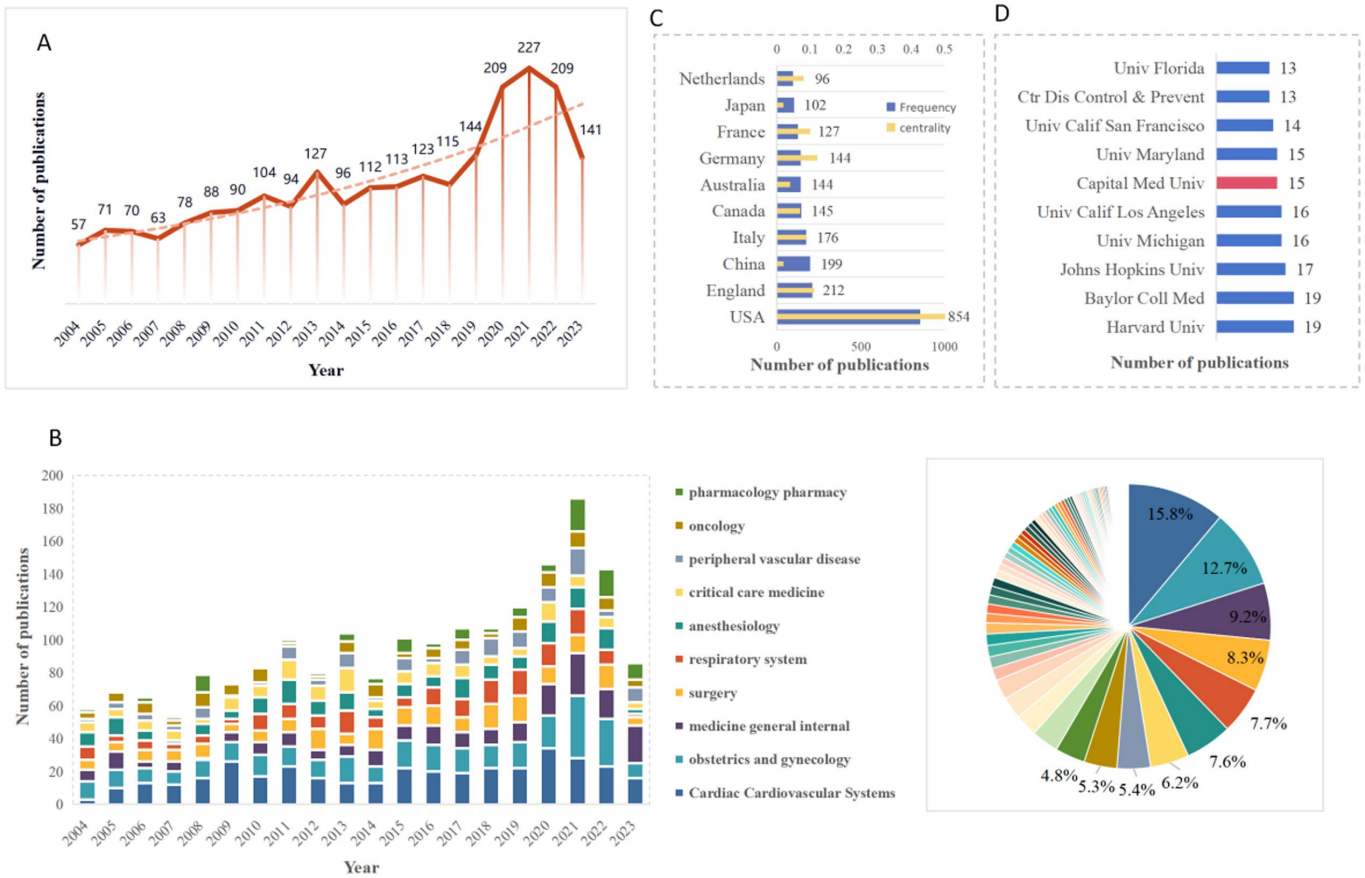
**(A)** The annual number of publications on the topic of peripartum respiratory complications from 2004 to 2023. **(B)** The annual number of publicaitons and the distribution of the top 10 subject categories. **(C)** The number of publicaitons and centrality of top 10 countries/regions. **(D)** The number of publicaitons of top 10 institutions.

The number of annual publications per year is a vital indicator reflecting the status and trends within a specific research field. The trend of annual paper publication is illustrated in Figure 2A, depicting a consistent increase with mild fluctuations from 2004 to 2018. As the theoretical foundations continue to solidify, the research on respiratory complications in pregnancy has entered a stable development stage. Between 2019 and 2022, there was a significant surge in articles within this field, with the majority published in 2020 (*n* = 227), potentially linked to the global COVID-19 epidemic. Concurrently, respiratory complications in pregnancy have garnered increased attention. The anticipation is that research in this field will further expand with a deepening understanding of various diseases.

Next, to determine the disciplines involved, we analyzed the subject categories in this field using Web of Science and presented the results in Figure 2B. The most predominant category was Cardiac Cardiovascular Systems (15.8%), followed by Obstetrics and Gynecology (12.7%). Other disciplines included Medicine General Internal (9.2%), Surgery (8.3%), and Respiratory System (7.7%). Additionally, Anesthesiology, Critical Care Medicine, Peripheral Vascular Disease, and Oncology accounted for a quarter of all papers, marking a notable aspect of research on respiratory complications in pregnancy.

In the initial phases of research, the investigation of respiratory complications in pregnancy was confined to the field of obstetrics and gynecology. However, as various disciplines have progressed and a more comprehensive systems thinking approach has been embraced in medicine overall, the scope of the condition has broadened. Consequently, an increasing number of disciplines are directing their attention toward understanding and addressing respiratory complications in the pregnant population.

### 3.2 Collaborative network analysis

#### 3.2.1 Countries/regions and institution analysis

Figures 2C,D illustrated the top 10 countries/regions and institutions based on publication numbers. Over the period from 2004 to 2023, 103 countries/regions published papers on respiratory complications during pregnancy, involving 564 institutions. The United States stood out as the most productive country with 854 publications, significantly surpassing the second-ranked China (*n* = 199), followed by the United Kingdom (*n* = 212). Notably, nine out of the top 10 institutions were affiliated with the United States, while the remaining one was from China. The leading institutions were Harvard University (*n* = 19), Baylor College of Medicine (*n* = 19), and Johns Hopkins University (*n* = 17).

Additionally, the United States held the highest betweenness centrality score (*n* = 0.52), indicating its pivotal role in cooperative links between countries and its leadership in research within this field. Germany and the United Kingdom also exhibited high centralities, both exceeding 0.1, highlighting their significant roles in international exchange. However, despite China’s substantial contributions to publications in this field, there is room for improvement in international collaboration, as evidenced by its relatively low betweenness centrality (0.02).

**Figure 3:**
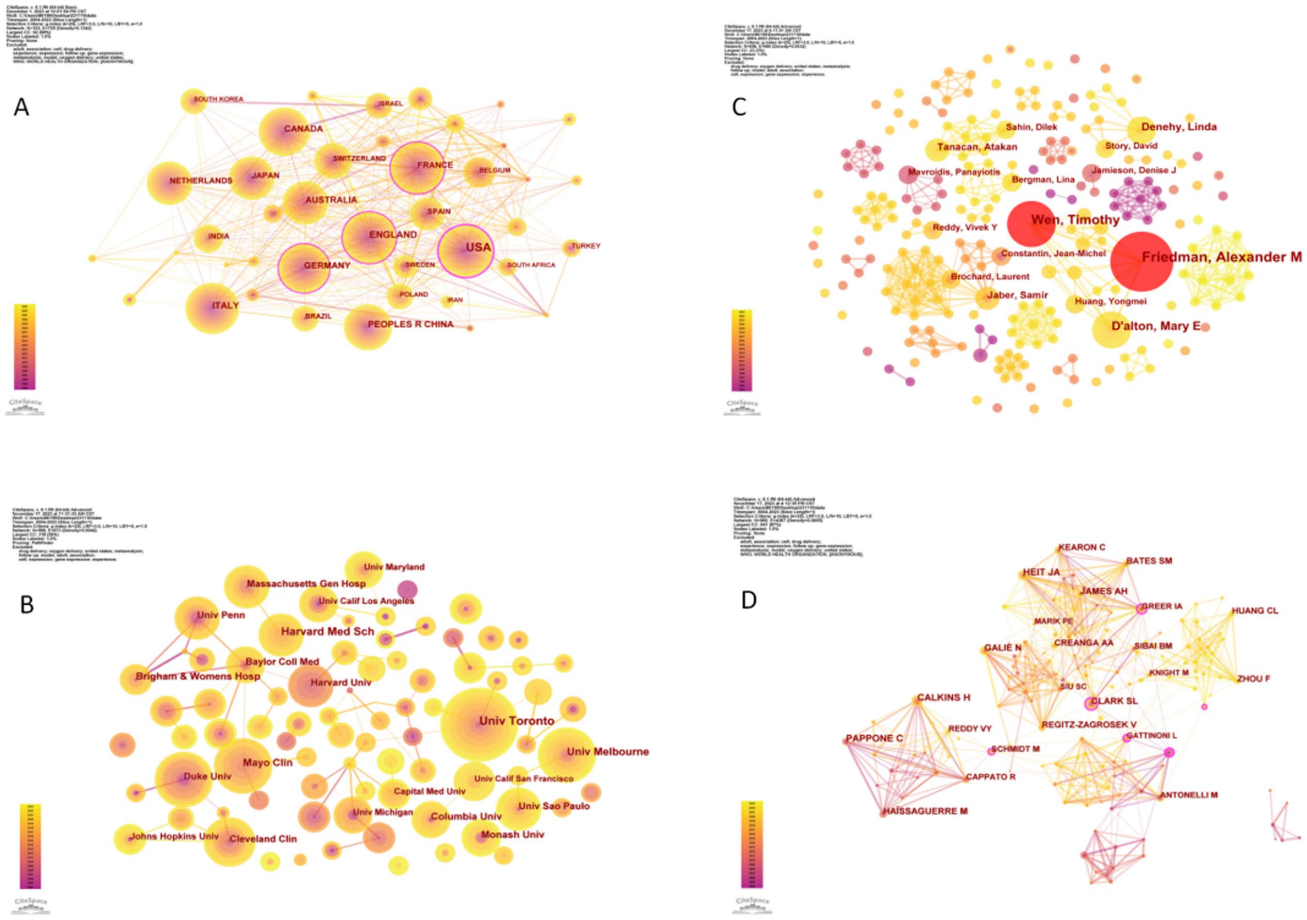
**(A)** A visualization of the country collaboration network. **(B)** A visualization of the institution collaboration network. **(C)** A visualization of the author collaboration network. **(D)** A visualization of the cited authors collaboration network.

The visual analysis of countries/regions and institutions was presented in Figures 3A,B. The merged network of co-country/region consisted of 103 nodes and 705 links, with a density of 0.13. In contrast, the co-institution network map comprised 568 nodes and 1,131 links, yielding a lower density of 0.007. This suggested that strong connections between other institutions have not been fully established. Consequently, it was anticipated that large-scale academic forums and academic visits should be organized to promote exchanges and cooperation among countries and institutions. Such initiatives have the potential to break down academic barriers, strengthen interdisciplinary collaboration, and foster integration across different fields.

#### 3.2.2 Authors and co-cited authors analysis

Friedman, Alexander M (*n* = 13), with the most substantial nodes in Figure 3C, emerged as the most active author in the field. Hailing from the Department of Obstetrics and Gynecology at Columbia University Vagelos College of Physicians and Surgeons, Friedman was closely followed by Wen, Timothy (*n* = 10), affiliated with the Department of Obstetrics, Gynecology, and Reproductive Sciences at the University of California, San Francisco. The red nodes highlighted authors who published prolifically within a short timeframe.

The co-cited authors network, depicted in Figure 3D, comprised 960 nodes and 4,367 links. The size of the nodes reflected the number of citations, with top-cited authors including Calkins, H (60 times), Heit, JA (56 times), and Pappone, C (51 times). Nodes marked with a purple circle, such as Gattinoni, L, Schmidt, M, Greer, IA, Creanga, AA, and Clark, SL, indicated high centrality, signifying their position between different groups of nodes (10).

### 3.3 Keyword analysis

#### 3.3.1 Keyword co-occurrence analysis

**Figure 4:**
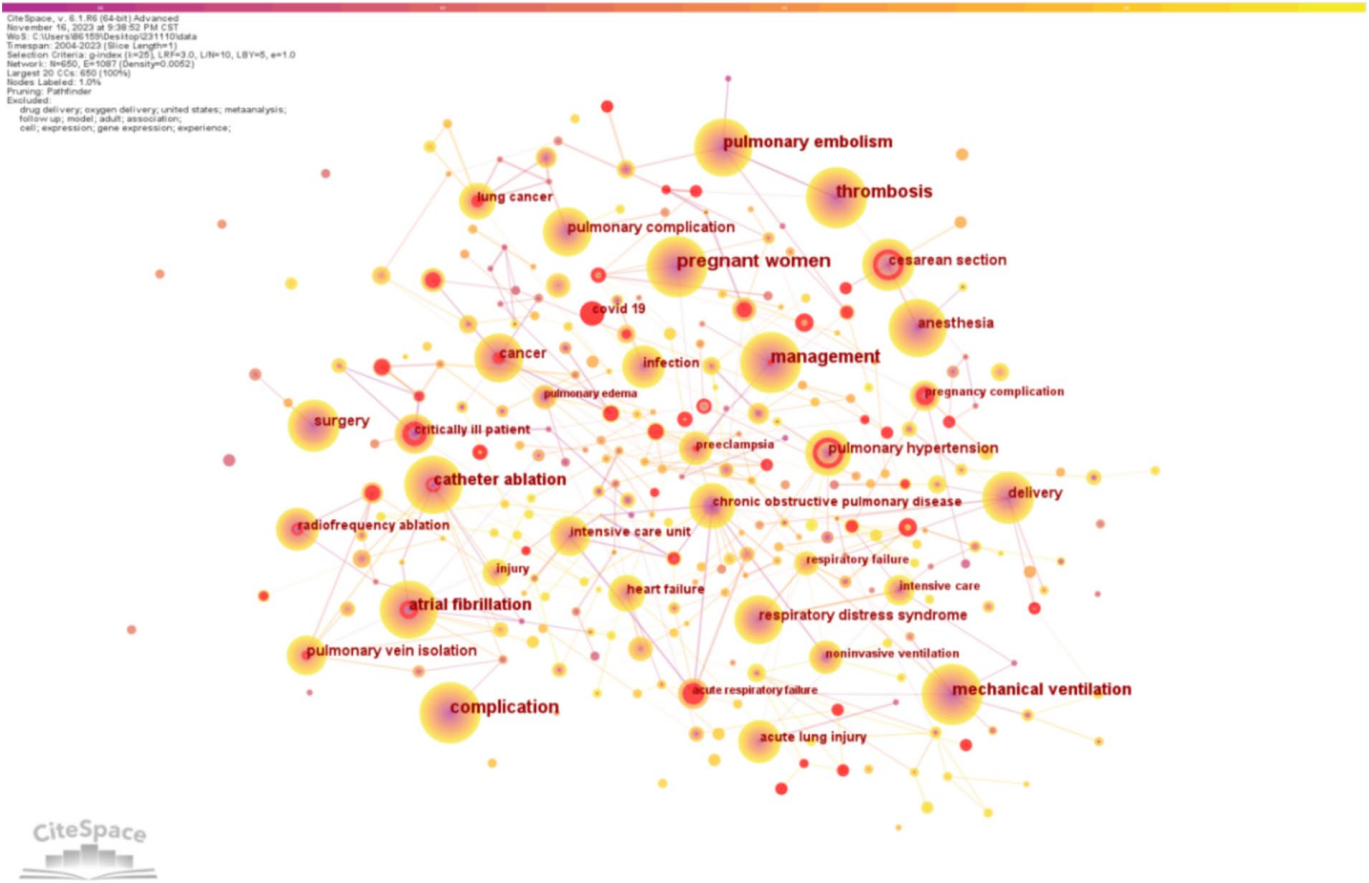
A visualization of the keywords network.

Keywords serve as an expression of the core content within the literature. Through the analysis of keyword frequency and clusters, we gain insights into the developmental trends and research hotspots in the field. As depicted in Figure 4, aside from keywords like “pregnant women” (*n* = 297), “management” (*n* = 245), and “complication” (*n* = 238), high-frequency keywords in this study included “thrombosis” (*n* = 198), “pulmonary embolism” (*n* = 144), “atrial fibrillation” (*n* = 142), “catheter ablation” (*n* = 121), “mechanical ventilation” (*n* = 116), “anesthesia” (*n* = 104), “surgery” (*n* = 98), “cesarean section” (*n* = 88), “pulmonary hypertension” (*n* = 85), and “respiratory distress syndrome” (*n* = 83).

#### 3.3.2 Keyword clustering analysis

**Figure 5:**
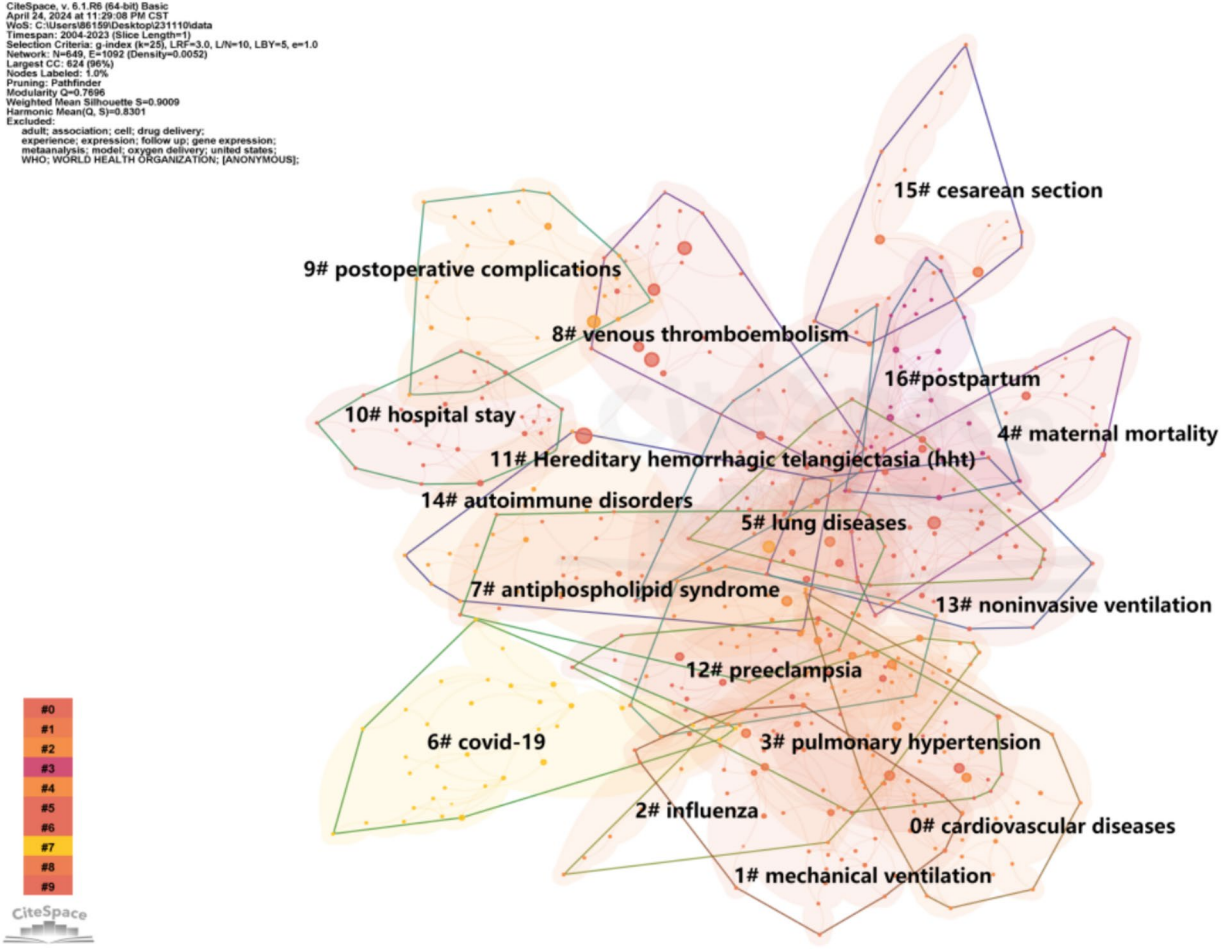
The clustered network map of keywords by using CiteSpace software.

In the following analysis we classified the total keywords into 17 clusters (#0 to #16). For conciseness only the information for the largest connected component was presented in Figure 5 while detailed information was listed in Table 1. Notably the silhouette value for each cluster was greater than 0.8 indicating the reliability of the clustering results.

**Table 1:** Summary of the largest 17 clusters of keywords.

Cluster ID	Size	Silhouette	Mean (year)	Label	Top terms
0	47	0.957	2011	Cardiovascular diseases	Cardiovascular diseases; atrial fibrillation; heart failure; congenital heart disease; pulmonary vein; cardiac outcome; preeclampsia; fistula
1	47	0.865	2011	Mechanical ventilation	Mechanical ventilation; respiratory failure; critical care; respiratory distress syndrome; extracorporeal membrane oxygenation
2	39	0.881	2013	Influenza	Influenza; H1N1; antiviral agents; vaccination; COVID-19; delivery; repair; SARS-CoV-2
3	37	0.905	2013	Pulmonary hypertension	Pulmonary hypertension; congenital heart disease; pulmonary arterial hypertension; heart failure; transplantation; pulmonary embolism; congenital heart disease
4	36	0.883	2008	Maternal mortality	Maternal mortality; care; morbidity; maternal death; pregnancy complications; risk pregnancy; pulmonary artery; epidural analgesia; management; cesarean section; bupivacaine; morphine
5	35	0.824	2011	Lung diseases	Lung diseases; pulmonary complication; lung injury; chronic obstructive pulmonary disease; acute respiratory failure; endotracheal intubation; intensive care unit; ventilation; pulmonary function; lung cancer
6	34	0.981	2018	COVID-19	SARS-CoV-2; COVID-19; NRF2; acute lung injury; acute respiratory failure; mechanical ventilation; extracorporeal membrane oxygenation; cytokine storm
7	34	0.910	2010	Antiphospholipid syndrome	Antiphospholipid syndrome; ovarian stimulation; burden; endothelial dysfunction; Hellp syndrome
8	33	0.983	2011	Venous thromboembolism	Venous thromboembolism; pulmonary embolism; deep vein thrombosis; factor v leiden; thrombophilia; venous thrombosis; deep venous thrombosis; pregnancy; risk factor
9	29	0.954	2012	Postoperative complications	Postoperative complications; risk; respiratory depression; postthrombotic syndrome; intrathecal morphine; pregnancy
10	26	0.909	2010	Hospital stay	Hospital stay; pulmonary artery catheter; randomized controlled trial; oxygen delivery; goal directed therapy; high-risk surgical patients
11	26	0.933	2013	Hereditary hemorrhagic telangiectasia (hht)	Hereditary hemorrhagic telangiectasia (hht); right heart failure; pharmacokinetics; survival; placental macrophages; transcatheter emboli
12	26	0.864	2016	Preeclampsia	Preeclampsia; hypertensive disorders; pulmonary edema; management; therapy; eclampsia; hypertensive crisis; atrial fibrillation
13	25	0.840	2012	Noninvasive ventilation	Noninvasive ventilation; acute respiratory failure; pregnancy; chronic obstructive pulmonary disease; general anesthesia; antiphospholipid syndrome
14	25	0.922	2015	Autoimmune disorders	Management; diabetes mellitus; autoimmune disorders; bronchiectasis; omalizumab; cardiovascular diseases
15	22	0.974	2013	Cesarean section	Cesarean section; cesarean delivery; spinal anesthesia; epidural anesthesia; general anesthesia
16	22	0.874	2015	Postpartum	Postpartum; atrial fibrillation; pulmonary embolism; deep vein thrombosis

Cluster #0, labeled as “cardiovascular diseases,” stands out as the predominant cluster. The occurrence of arrhythmias during pregnancy, particularly atrial fibrillation (AF) and ventricular tachycardia (VTA), is on the rise, presenting a potential or direct risk factor for cardiovascular-related mortality in expectant mothers. Besides, pregnant individuals experiencing arrhythmias or peripartum cardiomyopathy should carefully consider the heightened risk of heart failure, Intracardiac thrombosis and pulmonary embolism (11, 12).

Cluster #1 (mechanical ventilation) and cluster #13 (noninvasive ventilation) are concentrated on the study of ventilation therapy for acute respiratory failure (13, 14) and chronic obstructive pulmonary disease (15) in pregnant patients, incorporating the use of extracorporeal membrane oxygenation (16).

Cluster #2 (“influenza”) conducted research on the clinical characteristics and treatment of pregnant and postpartum women who were affected by influenza, including strains such as H1N1 (17).

Cluster #3 (“pulmonary hypertension”) explored a prevalent clinical disease wherein patients face an elevated risk of serious cardiovascular events during pregnancy or childbirth (18, 19).

Clusters #5 (“lung diseases”) is centered on the diagnosis, treatment, and prognosis of lung diseases, with a particular emphasis on those that are newly developed during pregnancy or were diagnosed before pregnancy, such as chronic obstructive pulmonary disease, asthma, and lung cancer (20).

Cluster #6, centered around “COVID-19,” concentrates on the clinical characteristics and outcomes of pregnant women affected by the coronavirus disease 2019 (21). The SARS-CoV-2 virus, responsible for causing COVID-19, exhibits strong infectivity and can trigger a cytokine storm, leading to acute respiratory distress syndrome (ARDS)-like lung damage. This can progress to pneumonia and severe lung injury, posing a high mortality risk in pregnant women (7, 22).

Cluster #7 (antiphospholipid syndrome) and Cluster #14 (autoimmune disorders), provided valuable insights into the pulmonary manifestations and pregnancy complications associated with antiphospholipid syndrome (23, 24). Furthermore, it highlights the serious consequences of some rare autoimmune diseases, such as systemic lupus erythematosus (SLE).

Cluster #8 (“venous thromboembolism”) delved into the study of venous thromboembolism, a critical challenge in maternal health. This encompasses two components, namely deep venous thrombosis (DVT) and pulmonary embolism (PE) (25). Hence, their risk factors, prevention strategies, and management during pregnancy and postpartum period were researched (26).

Cluster #9 (postoperative complications) concentrated on a range of postoperative pulmonary complications, including respiratory failure, pneumonia, acute respiratory distress syndrome, atelectasis, hypoxemia, and pulmonary embolism (27, 28).

Cluster #10 (“hospital stay”) primarily focuses on the treatment and care of postpartum women hospitalized due to respiratory complications, or respiratory complications occurring during the hospital stay.

Cluster #11 (hereditary hemorrhagic telangiectasia) concentrated on exploring the complications experienced by pregnant women with HHT, a dominantly inherited genetic vascular disorder (29).

Cluster #15 (“cesarean section”) was linked with venous thromboembolism (26). Furthermore, the choice of anesthesia methods and intraoperative airway management during cesarean sections may give rise to respiratory complications (30, 31).

Cluster #16 (postpartum) focused on the respiratory complications occurring in postpartum (32). Cluster #15 (preeclampsia) conducted research on preeclampsia, a significant cause of hypertensive acute pulmonary edema in pregnancy (33).

#### 3.3.3 Keyword burst analysis

**Figure 6:**
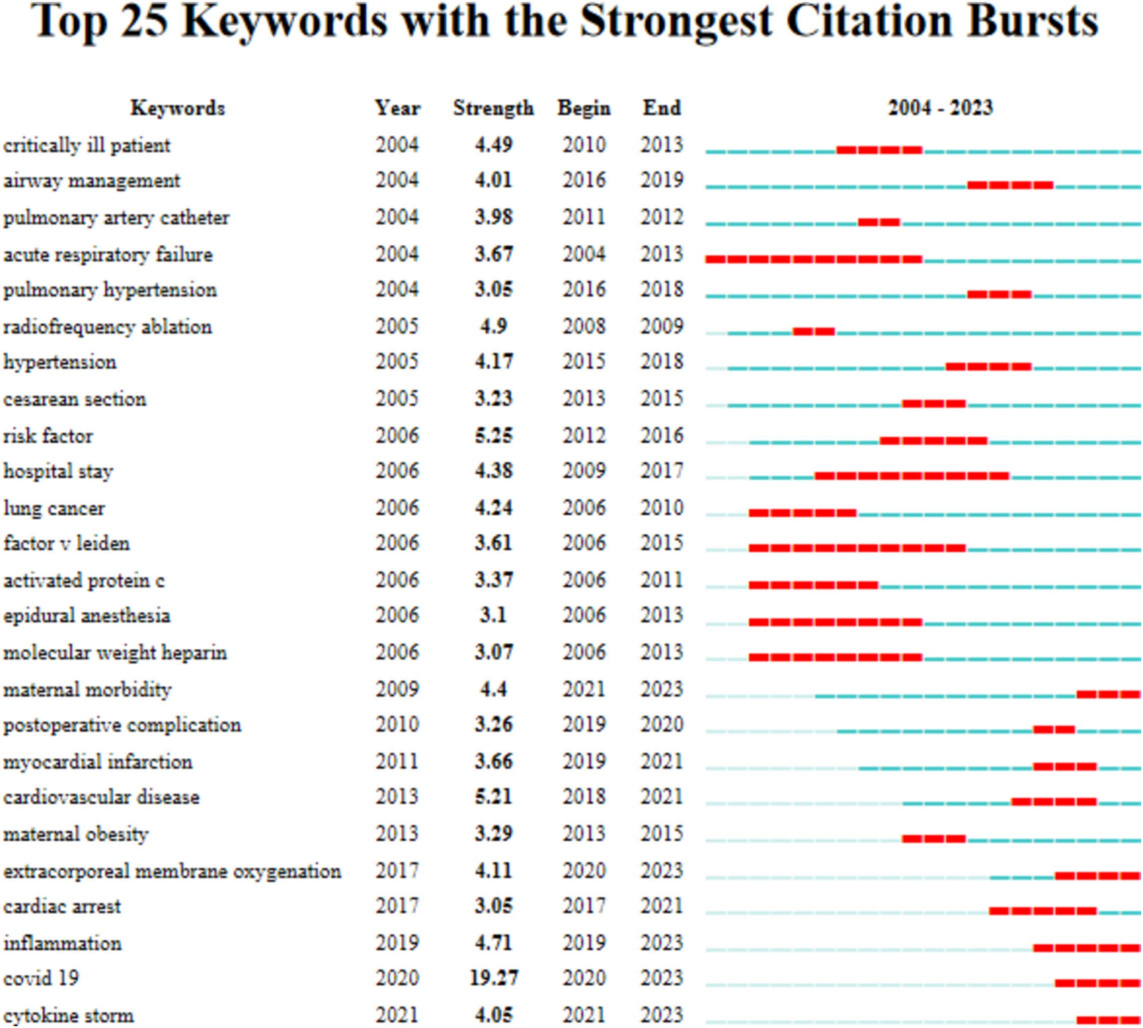
The top 25 keywords with the strong citation bursts between 2004 to 2023.

The top 25 keywords with citation bursts were presented in Figure 6, and the blue horizontal line delineates the duration of appearance for the identified keyword, while the red line signifies the specific period of its prominence or surge.

Over the past 5 years, burst keywords with a significant surge in citations include “COVID-19,” which has the strongest burst (*n* = 19.27) appeared in 2020, and “inflammation,” “extracorporeal membrane oxygenation,” “cytokine storm,” as well as “maternal morbidity.”

### 3.4 Literature co-citation and burst analysis

#### 3.4.1 Literature clustering analysis

**Figure 7:**
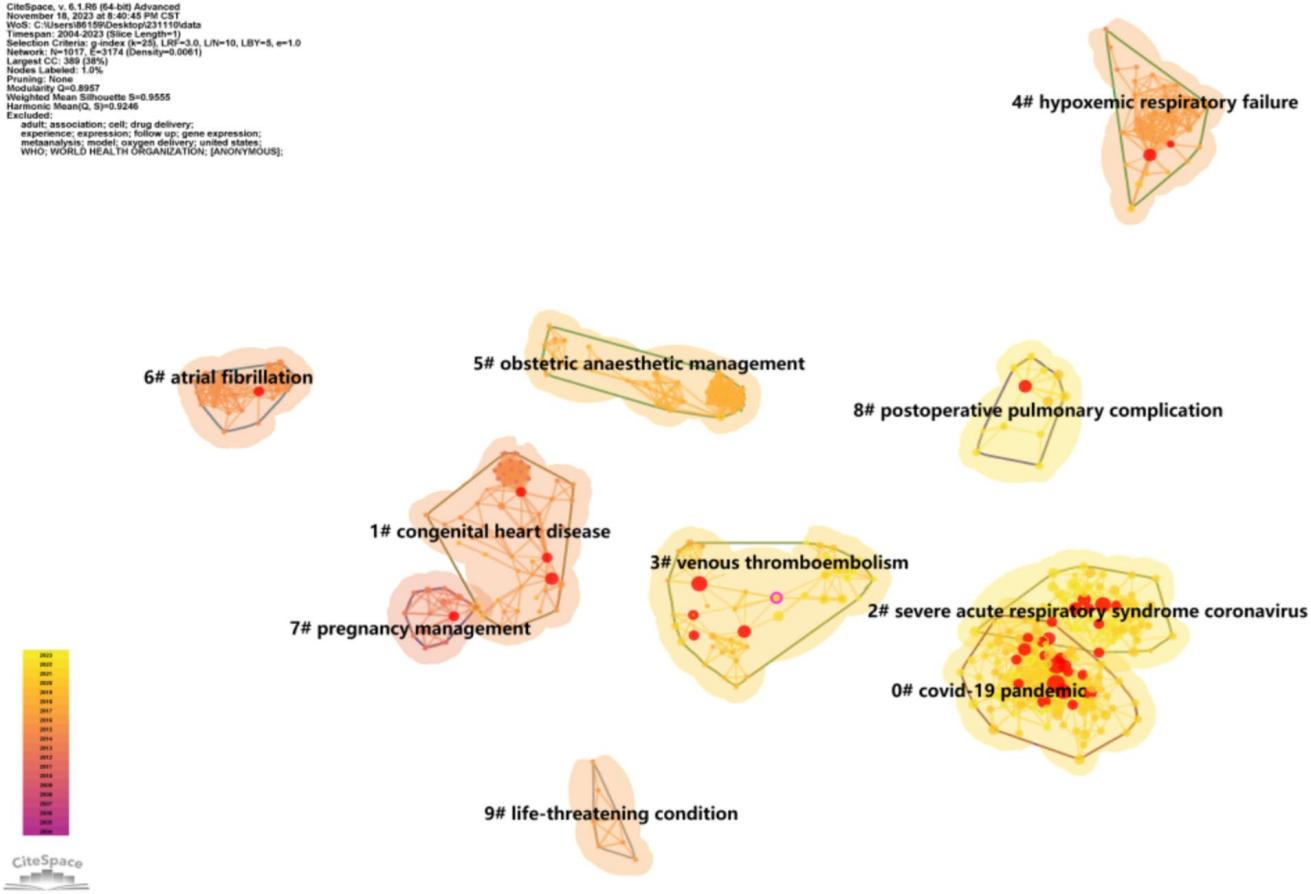
The clustered network map of reference by using CiteSpace software.

In this investigation, the decadal ranking of most cited references was delineated in Table 2. Furthermore, Figure 7 presented a clustered network diagram, derived from the bibliographies of 2,331 documents within the research domain of this study, revealing 10 pivotal clusters for detailed observation. The 10 major clusters label included COVID-19 pandemic, congenital heart disease, severe acute respiratory syndrome coronavirus, venous thromboembolism, hypoxemic respiratory failure, obstetric anaesthetic management, atrial fibrillation, pregnancy management, postoperative pulmonary complication, life-threatening condition.

**Table 2:** Top 10 cited references of publications in peripartum respiratory complications.

Rank	Count	Centrality	Title	Journal	First author	Year
1	34	0.05	Clinical course and risk factors for mortality of adult inpatients with COVID-19 in Wuhan, China: a retrospective cohort study	Lancet	Zhou Fei	2020
2	33	0.01	Clinical features of patients infected with 2019 novel coronavirus in Wuhan, China	Lancet	Huang Chaolin	2020
3	22	0.01	Characteristics of and Important Lessons From the Coronavirus Disease 2019 (COVID-19) Outbreak in China:Summary of a Report of 72,314 Cases From the Chinese Center for Disease Control and Prevention	JAMA-Journal of the Amercian Medical Association	Wu Zunyou	2020
4	20	0.00	Clinical manifestations, risk factors, and maternal and perinatal outcomes of coronavirus disease 2019 in pregnancy: living systematic review and meta-analysis	BMJ-British Medical Journal	Allotey John	2020
5	19	0.00	Clinical Characteristics of Coronavirus Disease 2019 in China	the New England Journal of Medicine	Wei-Jie Guan	2020
6	17	0.02	2015 ESC/ERS Guidelines for the diagnosis and treatment of pulmonary hypertension	European Heart Journal	Nazzareno Galiè	2016
7	16	0.00	H1N1 2009 influenza virus infection during pregnancy in the USA	Lancet	Denise J Jamieson	2009
8	16	0.08	Incidence of thrombotic complications in critically ill ICU patients with COVID-19	Thrombosis Research	F A Klok	2020
9	15	0.01	Pregnancy-Related Mortality in the United States, 2011–2013	Obstetrics and Gynecology	Creanga Andreea A	2017
10	14	0.08	Antithrombotic Therapy for VTE Disease: CHEST Guideline and Expert Panel Report	Chest	Clive Kearon	2016

#### 3.4.2 Timeline analysis of literature clustering

**Figure 8:**
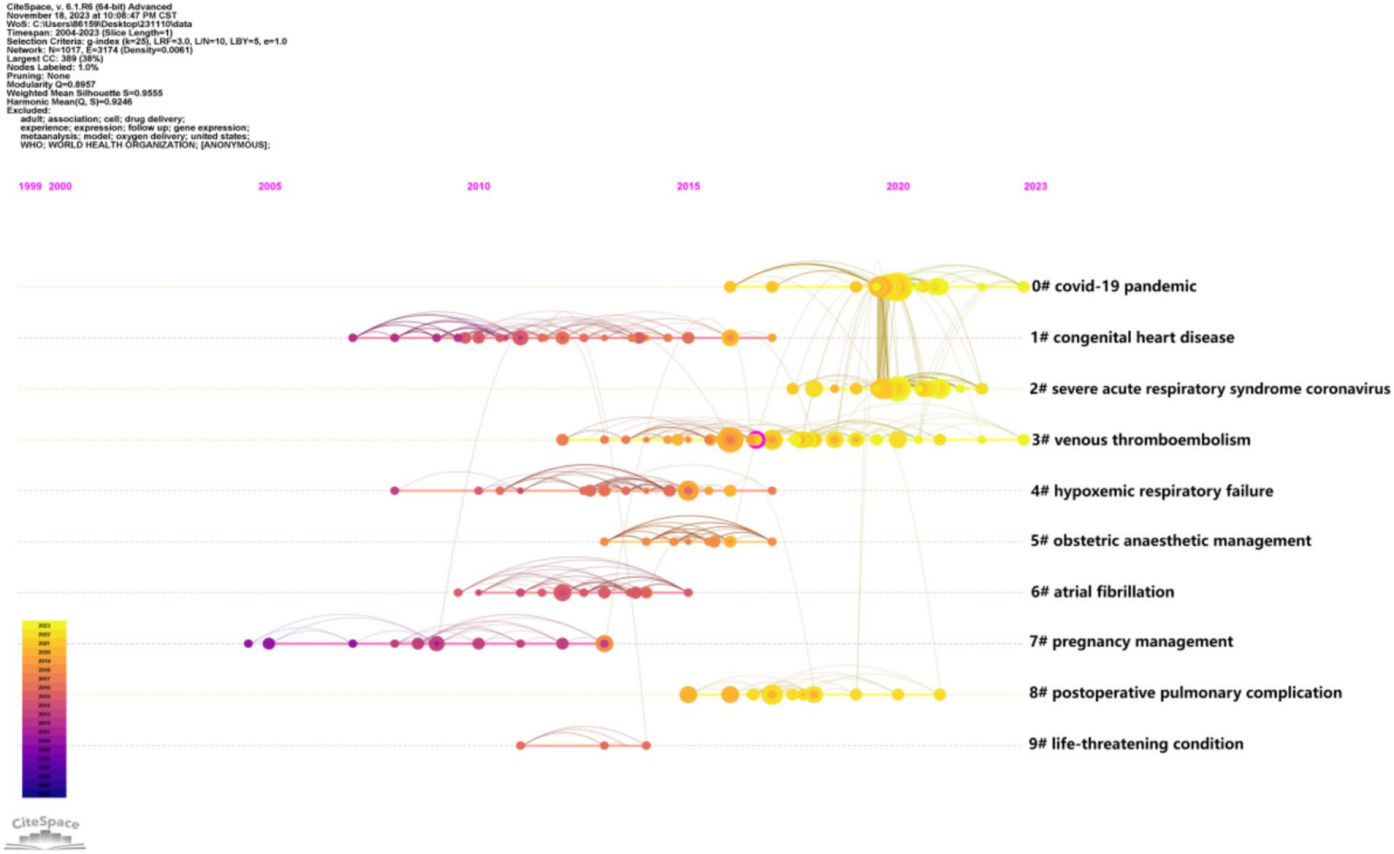
The timeline view of reference clusters by using CiteSpace software.

The alteration in color across individual clusters (shifting from purple to yellow) symbolizes the chronological evolution of the clusters (8, 9). Building upon this foundation, we conducted an analysis of the timeline view for the major clusters (Figure 8). In this figure, it was evident that before 2008, the research focus was primarily on pregnancy management. From 2008 to 2013, researchers predominantly delved into respiratory complications during pregnancy triggered by cardiovascular diseases. Over time, there has been a notable shift in attention toward respiratory complications induced by thrombotic disorders and obstetric anesthesia management (2014 to 2019). Recently, with the outbreak of the COVID-19 pandemic, the current research emphasis lies in acute respiratory failure caused by the coronavirus and post-cesarean pulmonary complications.

#### 3.4.3 Landmark references

**Figure 9:**
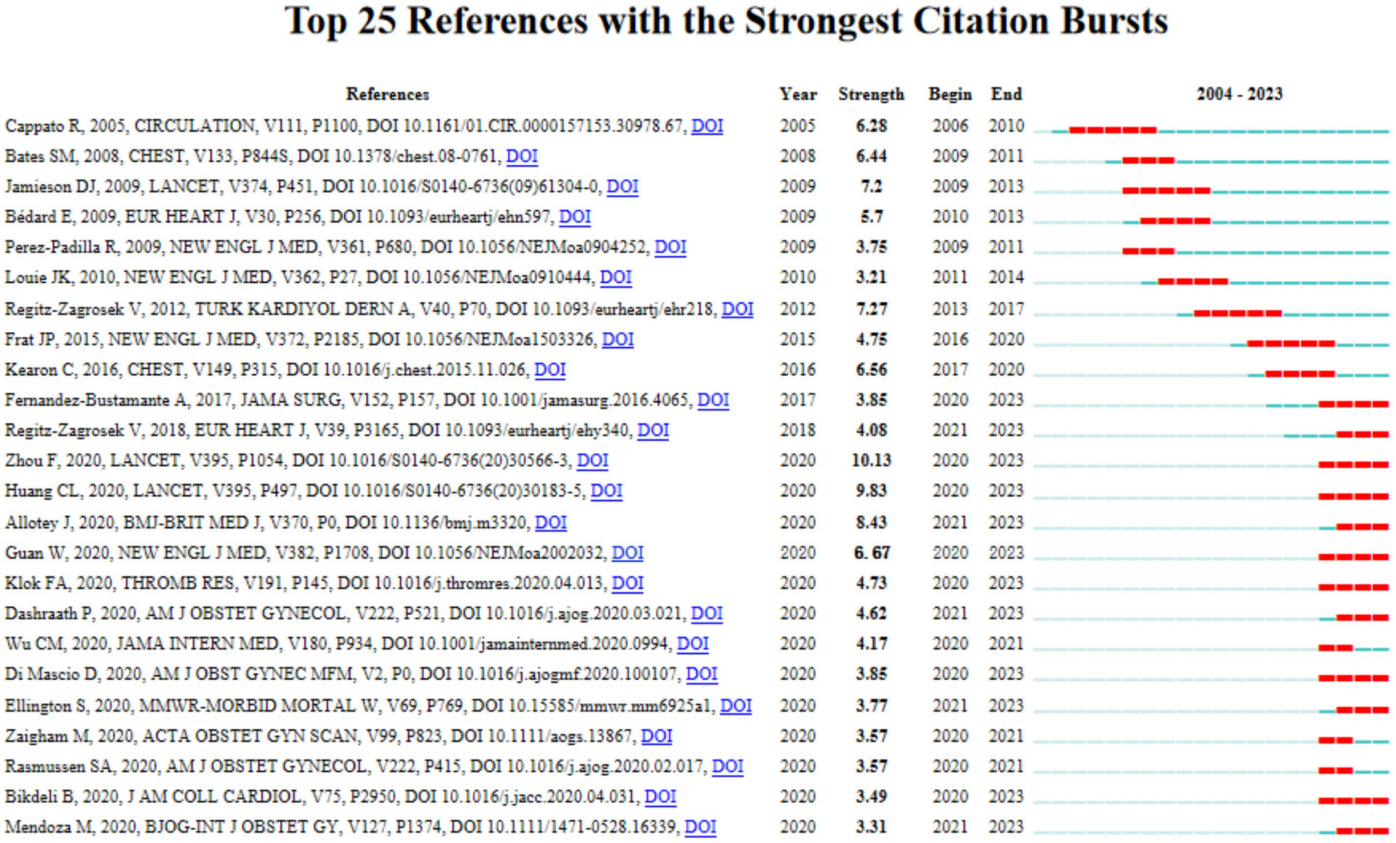
The Top25 reference with the strong citation bursts between 2004 to 2023.

In Figure 9, the top 25 references characterized by the strongest citation bursts was depicted, while Table 2 enumerated the top 10 cited references. Through a careful examination of these graphical representations, we can discern some literature of substantial significance.

The widespread outbreak of the novel coronavirus was the most prominent topic from 2019 to 2022, prompting an array of studies delving into its clinical characteristics, clinical prognosis, and treatment modalities. Notably, six out of the top 10 cited articles were related to this thematic domain.

### 3.5 Journal co-citation analysis

As listed in Table 3, Obstetrics and Gynecology emerged as the most prolific journal, boasting 30 publications. It was followed by the Journal of Interventional Cardiac Electrophysiology (*n* = 26), and Medicine (*n* = 25). Among these journals, the one with the highest Impact Factors (IF) were the Cochrane database of systematic reviews with an IF of 10.9. And the British Journal of Anesthesia followed with 9.6. It is observed that two journals were classified as Q3, and an additional one journal were classified as Q4. This classification implied that researchers in this field should strive to produce more articles of high quality.

**Table 3:** Top 10 co-cited journals and journals in peripartum respiratory complications.

Co-cited journal	Frequency	Impact factor (5 years)	JCR	Journal	Frequency	Impact factor (5 years)	JCR
New England Journal of Medicine	1,035	115.7	Q1	Obstetrics and Gynecology	30	7.6	Q1
Lancet	789	118.1	Q1	Journal of Maternal Fetal Neonatal Medicine	26	1.8	Q4
Chest	700	10	Q1	Medicine	25	1.9	Q3
JAMA-Journal of the American Medical Association	669	81.4	Q1	British Journal of Anaesthesia	25	9.6	Q1
Circulation	605	33.2	Q1	PloS One	23	3.8	Q2
Obstetrics and Gynecology	486	7.6	Q1	American Journal of Obstetrics and Gynecology	21	9.1	Q1
American Journal of Obstetrics and Gynecology	484	9.1	Q1	Anesthesia and Analgesia	20	5.7	Q1
Journal of the American College of Cardiology	447	25.3	Q1	Heart Rhythm	19	5.8	Q2
American Journal of Respiratory and Critical Care Medicine	416	21.9	Q1	Cochrane database of systematic reviews	19	10.9	Q1
PLoS One	392	3.8	Q2	International Journal of Obstetric Anesthesia	18	2.6	Q3

Among the co-cited journals, the New England Journal of Medicine (*n* = 1,035) emerged as the most frequently cited, followed by The Lancet (*n* = 789) and Chest (*n* = 700). Significantly, all three journals boasted Impact Factors (IF) exceeding 80, with The Lancet claiming the highest IF at 118.1, followed by the New England Journal of Medicine (IF = 115.7) and JAMA-Journal of the American Medical Association (IF = 81.4).

## 4 Discussion

We employed CiteSpace for bibliometric and visual analysis of articles related to respiratory complications during pregnancy. From 2004 to 2023, research in this field has expanded significantly, leading to an overall upward trend in annual publication volume. Notably, there was a surge in articles from 2019 to 2022, likely influenced by the major outbreak of the novel coronavirus in 2019.

Meanwhile, a simple disciplinary analysis reveals a diverse range of disciplines involved in researching this field. The predominant categories include Cardiac Cardiovascular Systems, Obstetrics and Gynecology, Medicine General Internal and Respiratory System. We also identified influential countries, research institutions, and key authors in the field and their collaboration network. Among the top 10 productive institutions, nine were from the USA and one was from China. The prolific institutions were Harvard University, Baylor College of Medicine, and Johns Hopkins University. The USA exhibited the highest publication volume and signifying its leadership role in this field and its essential position as a hub for collaboration with other countries.

Friedman Alexander M, affiliated with the Department of Obstetrics and Gynecology at Columbia University Vagelos College of Physicians and Surgeons, stands out as the preeminent contributor in this domain. A recent study (32) revealed that the discontinuity in postpartum care was associated with an increased risk of severe conditions, such as heart failure, thromboembolism, and upper respiratory tract infections, along with elevated costs and an extended duration of hospitalization. Several other studies demonstrated that pregnant women contracting influenza during childbirth hospitalization were at a higher risk of severe complications, such as mechanical ventilation and acute respiratory distress syndrome (34). Additionally, women with grand multiple pregnancies faced an increased risk of complications, including disseminated intravascular coagulation, pulmonary edema, and acute heart failure (35). Furthermore, asthma ranked among the most common complications during pregnancy and was associated with a series of moderate risks (including severe maternal morbidity, preeclampsia, gestational hypertension, postpartum hemorrhage, gestational diabetes, maternal mortality, and venous thromboembolism). However, no increased risk was found for severe respiratory system complications (mechanical ventilation, temporary tracheostomy, adult respiratory distress syndrome, and status asthmaticus) among deliveries with asthma (36).

As for the keyword clustering analysis, we classified the total keywords into 17 clusters. “Cardiovascular diseases” stands out as the predominant cluster. Peripartum cardiomyopathy and preeclampsia may lead to the development of complications such as disseminated intravascular coagulation syndrome, pulmonary edema, postpartum cerebrovascular diseases, and acute respiratory distress syndrome (37, 38). The occurrence of arrhythmias during pregnancy presenting a potential or direct risk factor for cardiovascular-related mortality in expectant mothers, which may increase the potential risk of pulmonary embolism. Pregnant women with pre-existing pulmonary arterial hypertension may suffer additional cardiac burden (39), which could trigger a series of respiratory complications, including shortness of breath, hypoxemia, and, in severe cases, respiratory failure (40, 41). “COVID-19” exhibits strong infectivity and can trigger a cytokine storm, leading to pneumonia, severe lung injury and acute respiratory distress syndrome (ARDS)-like lung damage, posing a high mortality risk in pregnant women. Due to the physiological changes during pregnancy, the symptoms of women who have pre-existing lung or cardiovascular diseases may worsen after pregnancy. Therefore, preconception counseling for such pregnant women is crucial, and the management of their respiratory tract should also be emphasized.

“Venous thromboembolism,” particularly “pulmonary embolisms” a critical challenge in maternal health. Factors related to pregnancy (cesarean section, preeclampsia, multiple pregnancies, gestational diabetes) and non-pregnancy-related factors (advanced maternal age, ethnicity, obesity, recent surgery, smoking, thrombophilia, *in vitro* fertilization) can increase the risk of venous thromboembolism (42). Autoimmune disorders like antiphospholipid syndrome, as well as Systemic lupus erythematosus (SLE) warrants attention. In addition to the increased risk of thrombotic events, antiphospholipid antibody syndrome can also directly lead to pulmonary manifestations, including pulmonary hypertension (as defined by echocardiography or cardiac catheterization), pleuritis, pulmonary hemorrhage, pneumonia, and pulmonary fibrosis. Recent studies have suggested that vitamin D may play a role in improving thrombotic events related to antiphospholipid antibody syndrome (43). Lupus myocarditis, the cardiac system complication of systemic lupus erythematosus, which presents as left ventricular dysfunction, acute heart failure, and pulmonary edema, is a rare but life-threatening complication that needs to be differentiated from pneumonia (44). Most pregnant patients with HHT may develop congenital pulmonary arteriovenous malformations, which can lead to low oxygen tolerance and increased risks of respiratory tract infections and thromboembolism, even life-threatening complications such as bleeding, massive hemoptysis, and hemothorax, requiring urgent surgical treatment.

Also, “cesarean section” was linked with venous thromboembolism (26). Furthermore, the choice of anesthesia methods and intraoperative airway management during cesarean sections may give rise to respiratory complications (30, 31). The top 25 keywords with citation bursts over the past 5 years were listed in our result, and “COVID-19” was the strongest burst appeared in 2020. As a result, scholars had shown extensive interest in understanding the pathogenic mechanisms of this disease and in managing pregnant women affected by it, with the goal of improving maternal survival rates. Therefore, the outbreak of the COVID-19 pandemic may be an important turning point, which shifted research focus away from areas like pulmonary hypertension, preeclampsia and arrhythmias, toward the infectious consequences of COVID-19, such as cytokine storm and hypercoagulability in pregnant women. Additionally, the treatment methods represented by ECMO may also be potential research hotspots in the near future.

The relationship in which two or more papers are simultaneously cited by one or more later papers is referred to as a co-citation relationship (45). CiteSpace facilitates the analysis of co-citation within scholarly literature. The amalgamation of co-cited documents shapes the foundational knowledge within a given domain, while the assemblage of citing documents that reference this knowledge base defines the cutting edge of research. The nomenclature assigned to clusters in CiteSpace is derived from the extraction of noun phrases found within the citing documents, thereby signifying the forefront of research in the respective field (8). In our investigation, we found 10 major clusters label included COVID-19 pandemic, congenital heart disease, severe acute respiratory syndrome coronavirus, venous thromboembolism, hypoxemic respiratory failure, obstetric management, atrial fibrillation, pregnancy management, postoperative pulmonary complication, life-threatening condition.

Study the landmark references, six out of the top 10 cited articles were related to coronavirus. “Clinical manifestations, risk factors, and maternal and perinatal outcomes of coronavirus disease 2019 in pregnancy: living systematic review and meta-analysis (46),” was published in BMJ-British Medical Journal by Allotey John in 2020. This analysis demonstrated that pregnant women and recently pregnant women are less likely to experience symptoms such as fever, respiratory difficulties, cough, and muscle pain compared to non-pregnant individuals with COVID-19. However, there is an increased risk of admission to the intensive care unit and receiving invasive ventilation. Advanced maternal age, high body mass index, non-white ethnicity, pre-existing comorbidities, and pregnancy-specific conditions (such as preeclampsia and gestational diabetes) are associated with severity.

Among the remaining four articles, one study (3) documented instances of H1N1 influenza virus infection in pregnant women during the 2009 outbreak in the United States. This article was published in the Lancet. Compared to non-pregnant patients, pregnant women were more prone to severe respiratory distress syndrome, necessitating urgent mechanical ventilation and exhibiting respiratory distress symptoms.

“2015 ESC/ERS Guidelines for the diagnosis and treatment of pulmonary hypertension” (47). This comprehensive guideline was published in the European Heart Journal in 2016. The guideline systematically elucidates the epidemiology and genetic research progress of pulmonary arterial hypertension, providing the basis for clinical diagnosis, such as clinical manifestations, electrocardiograms, chest X-rays, pulmonary function, arterial blood gas analysis, echocardiography, etc. It comprehensively describes the classification of pulmonary hypertension, including pulmonary arterial hypertension, pulmonary hypertension that due to left heart disease, pulmonary diseases and/or hypoxia, chronic thromboembolic pulmonary hypertension and pulmonary hypertension with unclear and/or multifactorial mechanisms.

## 5 Limitations

To ensure the quality of the articles, data were exclusively retrieved from the WoSCC database, potentially leading to the exclusion of papers not indexed in SCI. Continuous updates to the database emphasize the need for ongoing validation by future scholars in our field of analysis. Additionally, due to inherent limitations in CiteSpace software, the study focused solely on English literature, potentially overlooking relevant non-English specialized literature. Despite efforts to broaden the search strategy with extensive free terms, some articles pertinent to the research topic may not have been included. In summary, these limitations may introduce constraints to the comprehensiveness of our research results. However, the identified research trends in this paper are not expected to be significantly biased as a result of these constraints.

Besides, due to the COVID-19 outbreak from 2019 to 2022, research in this field has mostly focused on it in the past 3 years, potentially influencing future research priorities in the field. However, respiratory infections in pregnant women are already a significant concern, and lessons from COVID-19 may help us manage upper respiratory tract infections in critically ill pregnant women, reducing severe complications and improving outcomes.

## 6 Conclusion

This study undertook a bibliometric analysis of articles related to respiratory complications during pregnancy, specifically focusing on publications in SCI and ESCI journals over the last two decades. We utilized CiteSpace software to identify the primary countries, institutions, authors, and journals in this research field, offering potential collaboration references for interested researchers. Additionally, CiteSpace software was employed to expand the knowledge base in this field and analyze potential research hotspots.

Current research focuses on pregnancy-related venous thromboembolism and pulmonary embolism, as well as a series of preexisting diseases that may cause these clinical conditions. Additionally, there is attention to respiratory complications related to the impact of cardiovascular diseases represented by pulmonary hypertension and atrial fibrillation on the respiratory system, complications during surgeries including cesarean sections, anesthesia management, and postoperative respiratory complications.

## Data Availability

The original contributions presented in the study are included in the article/supplementary material, further inquiries can be directed to the corresponding authors.
